# Semen Quality in Rams Is Severely but Temporarily Affected by Bluetongue Virus Serotype 3 Infection

**DOI:** 10.3390/v17101371

**Published:** 2025-10-13

**Authors:** Ludovic Martinelle, Sophie Egyptien, Lola Dechene, Marielle Somville, Frédéric Derkenne, Stéfan Deleuze

**Affiliations:** 1 CARE-FEPEX Experimental Station, Fundamental and Applied Research for Animals & Health (FARAH), Faculty of Veterinary Medicine, University of Liège, 4000 Liège, Belgium; 2 Equine Theriogenology, Equine Clinical Sciences Department, Fundamental and Applied Research for Animals & Health (FARAH), Faculty of Veterinary Medicine, University of Liège, 4000 Liège, Belgium; segyptien@uliege.be (S.E.); msomville@uliege.be (M.S.); s.deleuze@uliege.be (S.D.); 3 Cabinet Vétérinaire de Julémont, 4650 Herve, Belgium; frederic.derkenne@hotmail.com

**Keywords:** Bluetongue virus, BTV-3, ram fertility, semen quality, reproductive virology

## Abstract

Bluetongue virus serotype 3 (BTV-3) emerged in northwestern Europe in 2023–2024, raising concerns about its potential reproductive impact on rams, similar to previous outbreaks with BTV-8. This study assessed the effect of natural BTV-3 infection on the semen quality of 49 rams in Belgium using two cross-sectional sampling sessions during the 2024 outbreak. Semen and blood were tested for BTV RNA via RT-qPCR, and a composite semen quality score (SQS) was established based on key sperm parameters. On the first sampling date, 75% of rams were viremic, and 19% presented azoospermia. Rams with BTV RNA detectable in both semen and blood had significantly lower SQS and sperm concentrations than those with viral RNA in blood only or none at all. By the second sampling, 53 days later, semen quality had improved markedly, indicating a transient effect of infection. These findings confirm that BTV-3 can severely but temporarily impair ram fertility, particularly when viral replication occurs in the reproductive tract. Given the seasonal overlap between vector activity and breeding programs, these results underscore the importance of integrating reproductive health monitoring into outbreak response strategies.

## 1. Introduction

Bluetongue is a non-contagious, vector-borne viral disease affecting domestic and wild ruminants, primarily sheep, cattle, and goats. Caused by the bluetongue virus (BTV), which belongs to the *Sedoreoviridae* family and *Orbivirus* genus, this disease is transmitted through bites from *Culicoides* midges [1,2]. The global distribution of bluetongue is closely linked to the presence of these vectors, with outbreaks occurring mainly in temperate and tropical regions [3]. Bluetongue has significant economic implications, primarily due to the loss of productivity, trade restrictions, and the associated costs of managing outbreaks. While sheep are often the most clinically affected, cattle serve as reservoirs of the virus, often remaining asymptomatic [4]. The virus is genetically diverse, comprising at least 36 distinct serotypes, including typical arthropod-borne serotypes 1 to 24, each varying in geographic distribution, virulence, and epidemiological impact [5,6].

In September 2023, BTV-3 was identified in the Netherlands [7,8]. The virus quickly spread throughout the country during the autumn of 2023. By the end of the year, several cases of BTV-3 had also been reported in Belgium, Germany, and the United Kingdom [8]. In Belgium, with the resumption of *Culicoides* vector activity in 2024, numerous BTV-3 outbreaks were daily detected since mid-July, affecting both cattle and sheep farms, and the disease gradually spread to the rest of the country.

Based on previous findings related to BTV serotype 8 (BTV-8) that emerged in the same area in 2006, the emergence of bluetongue virus serotype 3 (BTV-3) has raised concerns about its potential impact on ram fertility. Studies on BTV-8 have demonstrated that the virus can have detrimental effects on the reproductive health of rams, including a reduction in semen quality, testicular inflammation (orchitis), and subsequent infertility [9]. BTV-8 was shown to invade testicular tissue, potentially leading to persistent infections that impair sperm production and quality, with some cases exhibiting lasting damage to the reproductive system [10,11]. Given these findings, there is concern that BTV-3 may similarly target the reproductive organs, although specific research on BTV-3 reproductive effects remains limited so far.

Furthermore, the seasonality of bluetongue outbreaks, coinciding with the activity of Culicoides midges, can complicate breeding programs [3]. In many regions, breeding schedules are designed to maximize fertility during specific times of the year, aligning with market demands for lamb production. Bluetongue outbreaks during these critical breeding periods could severely impact ram fertility, leading to lower conception rates and suboptimal lambing outcomes [12].

Understanding the impact of BTV-3 on ram fertility is essential for developing targeted management and control strategies. In this study we report the impact of BTV-3 natural infection on semen quality of a total of 49 rams in Belgium using two cross-sectional analyses with partial repeated measures on a subset of the ram population.

## 2. Materials and Methods

### 2.1. Animal Selection

**Figure 1:**
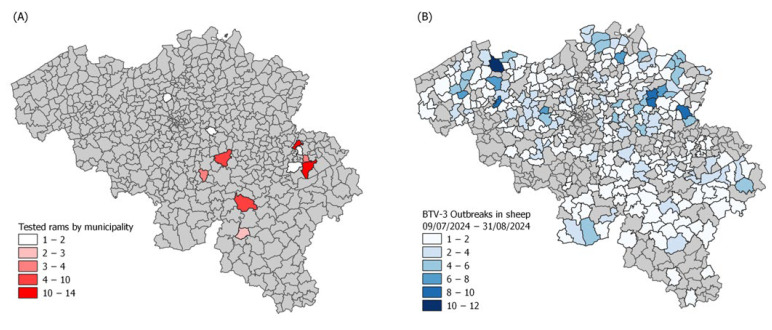
(**A**) Origin and number of tested rams by postal codes and (**B**) distribution of BTV-3 outbreaks in sheep from 9 July 2024 (first outbreak in cattle) to 31 August 2024 (D1) based on tests sent for clinical suspicion of BTV cases (Sciensano Institute, NRL for bluetongue disease, available at https://moriskin.sciensano.be/shiny/bluetongue/, accessed on 18 June 2025).

To be included in the study, rams had to be either fully vaccinated against BTV-3 with the last shot at least three weeks prior to testing or fully recovered from a natural BTV-3 infection for at least three weeks (based on farmers declarations). The vaccinated animals had to be fully vaccinated with one of the three available inactivated vaccines at the time in Belgium—Syvazul BTV-3 (Syva S.A.), BULTAVO 3 (Boehringer Ingelheim), or BLUEVAC-3 (CZ Vaccines S.A.U.)—and used on a voluntary basis to vaccinate cattle and sheep. These vaccines were not fully authorized for market placement but were granted an emergency use authorization under Regulation (EU) 2019/6, Article 110. Rams classified as recovered were declared by their owners as having shown clinical signs compatible with BTV-3 during the summer of 2024 and as being clinically healthy for at least three weeks before testing. This classification was further supported by the detection of anti-VP7 antibodies in all recovered rams and the absence of acute clinical signs at sampling. Rams’ breeds were distributed as follows: French Texel 26%, *Rouge de l’Ouest* 6%, *Bleu du Maine* 38%, *Dassenkop* 15%, *Vendéen* 6%, and Belgian Texel 9%. Samples were collected 50 days apart on two dates, the first one spanning the 31 August 2024 to 1 September 2024 (D1), and the second one on the 21 October 2024 (D2). A total of 49 rams were included; 36 were sampled on the first testing session and 18 on the second one, with 5 being tested on both sessions. The only partial overlap between samplings was due to logistical constraints (farmers availability and owners’ consent, as testing was voluntarily based) in the outbreak context. Rams originated mostly from the Walloon Region but also from Flanders (Figure 1A). D1 was 53 days after the first reported BTV-3 outbreak in 2024 in Belgium, which was chosen to approximately match the duration of one complete spermatogenic cycle in rams (about 47–48 days, [13]), thus allowing assessment of potential recovery after a full cycle (Figure 1B). Likewise, D2 was chosen 50 days after D1.

### 2.2. Sample Collection

#### 2.2.1. Blood Sampling

Each ram was blood sampled in a 9 mL dry tube and EDTA tube by jugular venipuncture. Blood samples were stored at 4 °C until further processing.

#### 2.2.2. Semen Collection and Analysis

Semen was collected into a pre-warmed collection vial using an artificial vagina in the presence of an in-estrus ewe. Semen evaluation was performed immediately after collection. The collected volume was recorded, and the gross appearance was classified as either viscous and creamy, aqueous and milky, or aqueous and translucent. After a 1:29 dilution in PBS, the concentration, total, and progressive motility were determined by Computer-Assisted Semen Analysis—CASA (Androscope ^®^, Minitube, Tiefenbach, Germany). A small volume (100 μL) was kept for further RNA extraction, and a small droplet was smeared on a slide for staining with Diff-Quick^®^ and morphology assessment. At least 100 spermatozoa were counted at microscopic magnification X1000, and the percentage of normal sperm cells with primary and secondary abnormalities was recorded. CASA in rams was used and validated by previous authors [14].

An overall semen quality score (SQS) ranging from 0 to 5 and accounting for the following semen traits—concentration, total motility, progressive motility, percentage of normal spermatozoa, and percentage of primary abnormalities—was determined using a method adapted from Leemans et al. (2012) [9]. Briefly, a score of ‘1’ was attributed to features satisfying the following conditions:

Sperm concentration > 1 × 10^9^/mL, total motility > 70%, progressive motility > 60%, percentage of normal spermatozoa > 70%, percentage of primary defect < 10%. The SQS was calculated for each ram by adding the scores assigned to each feature and ranged from 0 to 5. Volume was not included in the SQS calculation.

### 2.3. BTV RNA Detection

RNA extraction from EDTA blood and semen was achieved using the QIAamp Viral RNA Mini Kit (Qiagen, Hilden, Germany). Viral RNA denaturation, amplified segment, internal control, and real-time RT-PCR (RT-qPCR) by itself were the same as described by Toussaint et al. [15]. Briefly, the RT-qPCR targeted segment 5 with amplification performed on a Bio-Rad CFX96 thermocycler. The amplicon generated was 75 bases at the 5’ end of segment 5. This region is sufficiently conserved to allow hybridization of primers and probes to detect the 24 classical BTV serotypes 1 to 24. A Ct value < 40 was considered positive.

### 2.4. Anti-BTV Antibodies Detection

Anti-VP7 antibody detection in serum was performed by an accredited laboratory (ARSIA, *Association Régionale de Santé et d’Identification Animale*) using a commercial competitive ELISA kit (ID Screen^®^ Bluetongue Competition ELISA kit, ID Vet, France). This kit detects antibodies against the VP7 protein, which is group-specific and not serotype-specific. Thus, the kit is fully compatible with BTV-3 detection at the group level, although it cannot distinguish between serotypes. Results were expressed as % negativity (PN) compared to the negative kit control and transferred to a positive, doubtful, or negative result according to the cut-off settings provided by the manufacturer (PN  ≤  35 is positive; 35  <  PN  ≤  45 is doubtful; PN  >  45 is negative) as previously described [16].

### 2.5. Data Analysis

Correlation between blood and semen Ct values was analyzed using Spearman’s rank correlation test. Regarding rams tested on both D1 and D2, Ct variation in blood was assessed using the paired t-test. To allow for further analysis, a BTV-3 detection status score was set as 0 when semen and/or blood were RTqPCR negative and 1 when both semen and blood were RTqPCR positive. The association between the BTV-3 detection status score and SQS was tested using the Mann–Whitney test.

Spermatozoa concentrations of BTV RNA-fully negative animals, only blood-positive and both blood- and semen-positive animals were compared using the Kruskal–Wallis test and Dunn’s multiple comparison post hoc test, assuming non-normal distributions. Most statistical tests were performed using GraphPad version 5.01 for Windows, GraphPad Software, San Diego, CA, USA, www.graphpad.com. The quantitative analysis between Ct values in semen and sperm concentration was performed using R version 4.3.1, R Core Team (2023). _R: A Language and Environment for Statistical Computing_. R Foundation for Statistical Computing, Vienna, Austria. Scatter plot and regression line were created using the ggplot2 package [17]. For all tests, *p* values < 0.05 were considered significant.

## 3. Results

### 3.1. Animal Selection

Table 1 shows the rams tested on D1 in relation to their municipality of origin (based on the postal code) and time from the first reported BTV-3 outbreak in the respective municipalities (Sciensano Institute, NRL for bluetongue disease, available at https://moriskin.sciensano.be/shiny/bluetongue/, accessed on 18 June 2025), along with their serological status and RNA detection in blood and semen.

### 3.2. BTV RNA Detection

On D1, BTV RNA could be found in the blood of 27 out of 36 sampled rams (75%). Mean Ct was 28.91 (CI(95%) = (27.87–29.95)), whereas the median Ct value was 28.90. By contrast, BTV RNA could only be found in 14 semen samples (mean Ct 30.82 (CI(95%) = (29.07; 32.57)) and median = 31.15). All positive semen samples were associated with positive detection in blood. No correlation could be found between blood and semen Ct values (Spearman’s rank correlation, *p* > 0.8). Out of the 26 vaccinated rams (as per the declaration of the owners), 17 were found RTqPCR BTV positive at least in blood (65%), including 10 that were positive in both blood and semen (38.5%).

On D2 BTV RNA could be found in the blood of 15 out of 18 sampled rams (83.3%). Mean Ct was 28.41 (CI(95%) = (26.96–29.87)), whereas the median Ct value was 27.40. In semen, however, only one slightly positive sample could be detected (Ct = 37.9).

Ct values in the blood of rams that were tested on both dates (D1 and D2) did not differ significantly (paired *t*-test, *p* = 0.57).

### 3.3. Anti-BTV Antibodies Detection

All rams but one tested positive by ELISA on D1. The negative animal was, however, declared to be fully vaccinated and was negative in blood and semen at that time. Seven animals were declared to have recovered from natural BTV infection, whereas the remaining 29 rams were declared fully vaccinated.

Two rams tested negative on D2 and likewise were negative for BTV RNA in semen and blood. Both animals were declared to be fully vaccinated.

### 3.4. Semen Analysis

The expected normal sperm concentration during the breeding season is around 4000 × 10^6^ cells/mL, with a total motility of 90% and progressive motility of 40 to 45% [14].

On day 1 (D1), 7 out of 36 rams presented with azoospermia. Among the rams producing semen, the mean sperm concentration was 1067 × 10^6^ sperm/mL, with a total motility of 13.9% and progressive motility of 11.3%. Normal sperm morphology was observed in 32.5% of spermatozoa, with 10.9% exhibiting primary abnormalities and 40.7% secondary abnormalities.

On D2, 51 days after, the mean concentration was 750 × 10^6^ sperm/mL. Total motility averaged 36.6%, and progressive motility 32.5%. Normal sperm morphology increased to 72%, with 8.1% of primary anomalies and 19.7% of secondary anomalies. Notably, two rams exhibited numerous neutrophils on their semen smears.

**Figure 2:**
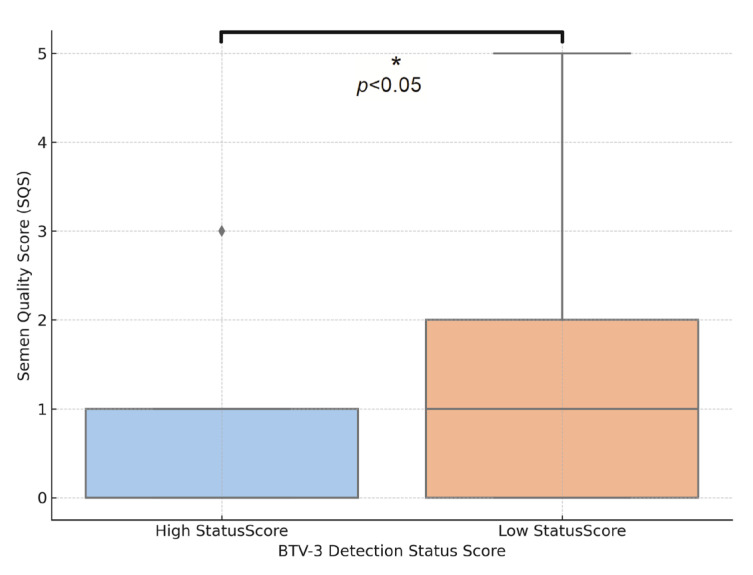
Comparison of semen quality score by BTV-3 RNA detection status score.

Figure 2 compares semen quality score (SQS) between two groups of rams categorized by their BTV-3 detection status (low status score: rams with BTV-3 RNA undetectable or detected in only one compartment (blood or semen) and high status score: rams with BTV-3 RNA detected in both blood and semen, indicating a higher systemic and reproductive tract viral load).

BTV-3 RNA presence in both blood and semen is associated with poorer semen quality (Mann–Whitney U test (*p* = 0.034).

**Figure 3:**
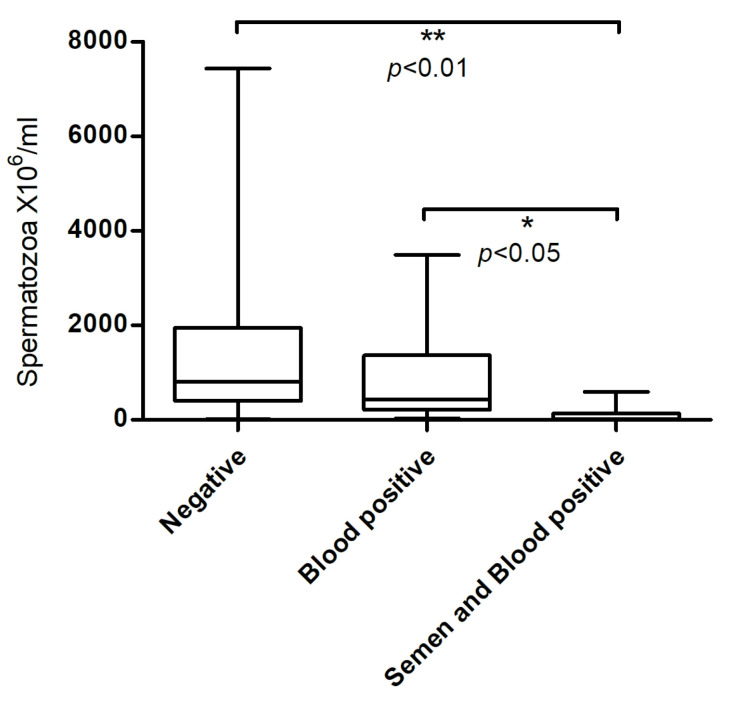
Sperm concentration by BTV-3 RNA detection in blood and semen.

This is further supported by the statistically significant difference in sperm concentration between rams positive for BTV in both semen and blood and those positive in blood only (Kruskal–Wallis test, *p* < 0.01), as well as between rams positive in both and those testing negative (Kruskal–Wallis test, *p* < 0.05; Figure 3).

**Figure 4:**
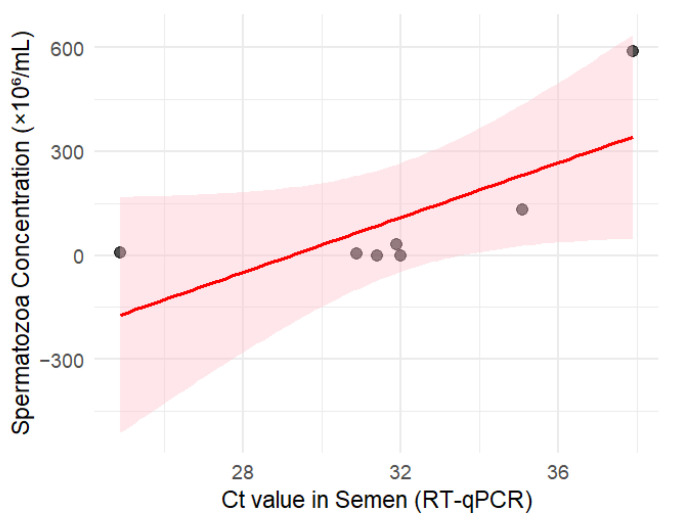
Semen Ct value vs. spermatozoa concentration. The pink shaded area corresponds to the 95% confidence interval.

Moreover, there is a moderate positive correlation between Ct value in semen and sperm concentration (Figure 4). This suggests that higher viral load (lower Ct) may be associated with reduced spermatozoa concentration. However, the association is not statistically significant, likely due to the small sample size (n = 7, Spearman’s rho (ρ) = +0.54, *p* = 0.21).

## 4. Discussion

The present study provides critical data on the impact of natural BTV-3 infection on ram fertility during the 2024 outbreak in Belgium. Indeed, the circulation of BTV-3 heavily impacted ruminant farming in Belgium in 2024, causing direct production losses, including abortions, premature births, and increased mortality of approximately 1% of the cattle population [18].

Our results contribute to the limited body of evidence regarding the reproductive consequences of BTV-3 in ruminants, particularly in male animals, and offer comparative insights relative to prior outbreaks caused by BTV-8. BTV’s known pathogenesis includes endothelial cell damage, viremia, and reproductive tissue tropism, as established in BTV-8 models [4]. Although systemic clinical signs such as fever could contribute to the impairment of semen quality, the reproductive dysfunction in rams is hypothesized to result from direct testicular invasion and localized inflammation (orchitis), possibly via systemic dissemination of the virus [19]. The study findings demonstrate a clear association between the presence of BTV-3 RNA in both blood and semen and a marked deterioration in semen quality, as assessed by a composite semen quality score (SQS). Rams with detectable viral RNA in both compartments (blood and semen) exhibited significantly reduced sperm concentration, motility, and morphological normality compared to rams with virus detected only in blood or not at all. This relationship aligns with earlier observations in BTV-8 infections, where Leemans et al. (2012) [9] reported a similar inverse correlation between viral RNA load and semen parameters, including concentration and morphology. The results obtained during the two cross-sectional analyses showed azoospermia in almost 20% of cases (7/36 rams) and severe oligospermia in 53% of cases (19/36 rams). Asthenospermia and teratospermia were also highly represented in animals tested at D1, with only 13.9% of progressive motility and 32.5% of normal sperm morphology, respectively. Secondary anomalies were more frequent than primary anomalies. By October 2024, progressive motility had increased to 32%, around three times higher than the initial result. Normal morphology had also improved to 72%. This is consistent with partial recovery after one spermatogenic cycle. The single semen sample testing weakly positive at D2 (Ct = 37.9) likely represents residual RNA, further supporting that viral shedding in semen is transient.

Comparable results have been described in bulls naturally infected with BTV-8 in Germany during the 2007 outbreak [20]. In that study, six PCR-positive bulls remained clinically asymptomatic but exhibited a transient impairment in semen quality. While sperm volume and concentration were not affected, post-thawing motility was significantly reduced in infected bulls (44.1% vs. 58.0% in controls, *p* < 0.001). Furthermore, the proportion of malformed sperm exceeded the 20% acceptable threshold during the viremic period, with tail abnormalities being the most common defects. Importantly, semen quality only gradually returned to normal after the clearance of detectable BTV RNA, highlighting that reproductive consequences can extend beyond the acute infection phase. These findings mirror our observations in rams, where semen abnormalities were most pronounced during viral RNA detection in blood and semen and where recovery was incomplete until at least one spermatogenic cycle had elapsed.

Virus isolation was not attempted in this study. This was primarily due to the emergency outbreak setting, limited resources, and biosafety considerations.

With a 50-day interval between the two samplings, the five rams available for comparison showed some improvement in sperm quality, particularly regarding sperm morphology.

The study convincingly demonstrates that BTV-3 infection is associated with a temporary but substantial impairment of ram semen quality, especially when the virus RNA is present in both blood and semen. Though fertility parameters improve within weeks, the initial damage could jeopardize breeding outcomes if not properly anticipated. These findings underscore the need for BTV-specific reproductive surveillance and breeding program adjustments during outbreaks.

## Figures and Tables

**Table 1:** Rams tested on D1, municipality of origin, time from first reported BTV-3 outbreak in the municipality of the ram to test (in days), declared vaccinated or recovered status of the animals, serology, and RNA detection in blood and semen.

Sample_ID	Postal Code	Date First Case	Time to Test	Age (Year)	Vaccinated/Recovered	Ct in Semen	Ct in Blood	ELISA
1	5190	12-08-24	19	1.5	Vaccinated	31.4	27.1	Pos
2	5190	12-08-24	19	0.5	Vaccinated	Neg	Neg	Pos
3	3640	19-07-24	43	1	Vaccinated	Neg	Neg	Pos
4	5190	12-08-24	19	0.5	Vaccinated	Neg	Neg	Neg
5	4650	19-07-24	43	1	Vaccinated	Neg	Neg	Pos
6	5190	12-08-24	19	1.5	Recovered	NA	26.6	Pos
7	3640	19-07-24	43	2	Vaccinated	Neg	34.3	Pos
8	6920	06-08-24	25	1.5	Vaccinated	Neg	31.4	Pos
9	1840	12-08-24	19	2	Vaccinated	Neg	Neg	Pos
10	1840	12-08-24	19	0.5	Vaccinated	26.7	28	Pos
11	6920	06-08-24	25	4.5	Vaccinated	30.9	28.7	Pos
12	6920	06-08-24	25	4.5	Vaccinated	Neg	25.2	Pos
13	5590	05-08-24	26	2	Vaccinated	26.6	28.9	Pos
14	4670	27-08-24	4	1	Vaccinated	34.9	34.3	Pos
15	5590	05-08-24	26	1	Vaccinated	31.2	32.9	Pos
16	4670	27-08-24	4	5	Vaccinated	Neg	29.7	Pos
17	4910	24-07-24	38	1.5	Vaccinated	34.1	28.4	Pos
18	4910	24-07-24	38	3.5	Vaccinated	31.1	30.2	Pos
19	4910	24-07-24	38	1.5	Vaccinated	Neg	25.3	Pos
20	4910	24-07-24	38	3	Vaccinated	29,8	28.3	Pos
21	4860	NA	NA	2	Vaccinated	NA	Neg	Pos
22	4910	24-07-24	38	NA	Vaccinated	Neg	Neg	Pos
23	4670	27-08-24	4	5	Vaccinated	Neg	28	Pos
24	4860	NA	NA	0.5	Vaccinated	Neg	Neg	Pos
25	4670	27-08-24	4	1	Vaccinated	Neg	29.3	Pos
26	4860	NA	NA	0.5	Vaccinated	Neg	28.9	Pos
27	4650	19-07-24	43	0.5	Vaccinated	Neg	NA	Pos
28	1367	01-08-24	30	2	Recovered	NA	23.5	Pos
29	5590	05-08-24	26	2	Recovered	30,9	31.1	Pos
30	5310	10-08-24	21	0.5	Recovered	31,9	25.5	Pos
31	5590	05-08-24	26	1	Recovered	Neg	31.7	Pos
32	5590	05-08-24	26	2	Recovered	Neg	29.2	Pos
33	5310	10-08-24	21	2.5	Recovered	35,1	26.9	Pos
34	1840	12-08-24	19	2	Recovered	24,9	28.5	Pos
35	4670	27-08-24	4	4	Recovered	32	28.9	Pos
36	5590	05-08-24	26	2	Recovered	Neg	29.8	Pos

No relationship was found between time to test and blood Ct values (Spearman correlation: ρ = 0.049, *p* > 0.8) and time to test and semen Ct values (Spearman correlation: ρ = −0.2, *p* > 0.48). The average time to test was 24.75 days (median = 25 days). One municipality (postal code 4860) had no BTV-3 outbreaks in sheep reported by the time of testing, only one cattle case reported on 23/08/2024 (8 days before testing). Three rams from that municipality were tested; all were declared to be vaccinated, but still one out of three was RTqPCR positive in blood. The other two rams were ELISA positive, but no viral RNA could be detected either in blood or semen. In addition, there was no significant difference between vaccinated and recovered animals regarding time to test (mean 21.63 and 25.83 for recovered and vaccinated animals, respectively, *p* > 0.66).

## Data Availability

Dataset available on request from the authors.

## Abbreviations

The following abbreviations are used in this manuscript:

ARSIA	*Association Régionale de Santé et d’Identification Animale*
BTV	Bluetongue Virus
CASA	Computer-Assisted Semen Analysis
Ct	Cycle Threshold
NRL	National Reference Laboratory
PN	Percentage of Negativity
SQS	Semen Quality Score
VP	Viral Protein
